# Inoculation of *Priestia megaterium* confers drought tolerance to garden pea (*Pisum sativum*)

**DOI:** 10.1038/s41598-026-48712-y

**Published:** 2026-04-13

**Authors:** Priyanka Khati, Pankaj Kumar Mishra, S. Santhiya, Rajendra Prasad Meena, Lakshmi Kant

**Affiliations:** ICAR Vivekananda Parvatiya Krishi Anusandhan Sansthan, Almora, Uttarakhand 263601 India

**Keywords:** Drought stress mitigation, *Pisum sativum*, PGP inoculants, Relative water content RWC, Na/K Ratio, Microbiology, Physiology, Plant sciences

## Abstract

A pot experiment was conducted to evaluate the potential of six PGP microbial isolates (FM20, FM65, FM19, W22, OF5, and Control) to mitigate the adverse effects of drought stress (40% FC) on pea (*Pisum sativum*) compared to normal irrigation (80% FC). The results demonstrated that PGP inoculants significantly enhanced physiological, ionic, and yield parameters under both normal and stress conditions consistently outperforming the uninoculated control. Under drought stress, W22 maintained the highest Relative Water Content (88.5%), statistically similar to several normal treatments, indicating superior water balance maintenance. In terms of ionic homeostasis, isolate FM20 was the most superior, achieving a 17.23% reduction in the detrimental Na/K ratio in straw compared to the stressed control and the largest reduction in Na-grain content (− 10.53%), confirming its role as the best absolute Na excluder. Under normal irrigation condition FM65 maximized both seed weight per plant (6.7 g) and 100 seed weight (22.9 g). On the other hand under drought (40% FC) OF5 yielded the highest seed weight per plant (7.3 g) and W22 produced the largest 100 seed weight (8.7 g). Collectively, the results confirm that PGP microbial inoculants, particularly FM65, OF5, and W22, offer a viable, strain-specific strategy to stabilize physiological functions, enhance nutrient utilization, and significantly alleviate drought-induced yield reduction in *P. sativum*.

## Introduction

Drought stress stands as the most critical abiotic constraint globally, severely limiting crop yield and threatening food security, with its intensity and frequency predicted to escalate due to climate change^1^. Legume crops, such as peas (*Pisum sativum* L.), are particularly vulnerable to water deficit during key developmental stages (e.g., vegetative and reproductive), leading to reduced germination, stunted growth, and significant yield losses^2^.

Traditional breeding for drought resistance is often time-consuming and costly. Consequently, the application of PGPR has emerged as a promising, sustainable, and cost-effective strategy to enhance plant resilience and productivity under water-limited conditions^3^. PGPR colonize the rhizosphere and plant roots, directly and indirectly modulating plant physiology to counteract the negative effects of drought. Their effectiveness is often amplified under stress conditions^4–8^.

The inoculation of PGPR has been consistently shown to induce key physiological changes in host plants, which are critical for survival and growth under water deficit. PGPR stimulate the accumulation of osmolytes, such as proline and soluble sugars, which maintain cell turgor and stabilize cellular membranes and proteins against dehydration^9–11^.

By maintaining better water potential and reducing oxidative damage, PGPR help sustain photosynthetic efficiency, leading to greater biomass and reproductive yield even under stress^2,8^.The bacterial genus *Priestia* (formerly a part of *Bacillus*) includes species like *Priestia megaterium*, which are widely recognized for their versatile biotechnological applications, including being potent PGPR. Recent genomic and experimental studies have solidified the role of *P. megaterium* in enhancing plant stress tolerance and growth promotion. *P. megaterium* strains have been shown to be effective bioinoculants in poplar seedlings demonstrating abilities of abiotic stress tolerance^12–15^.

The present investigation, which evaluates two *Priestia megaterium* strains (W22 and FM 65) alongside other multi-trait PGPR (OF5, FM20, and FM19) as reported by Khati et al.^16^, is consistent with the latest scientific trend of leveraging characterized, high-potential microbial strains to combat concurrent challenges of nutrient deficiency (Zn, P solubilization) and drought stress in essential crops like peas. While *Priestia megaterium* has been widely studied under controlled or seedling conditions still limited information exists regarding their combined role in drought mitigation and nutrient biofortification and their physiological modulation in peas under defined water deficit (40% field capacity). The selection of these strains, which demonstrated good PGPR traits (e.g., Zn/P solubilization, siderophore production, and PEG-6000 tolerance, according to the original work), provides a strong rationale for assessing their field potential in enhancing plant physiological status (RWC, proline content), yield components (pods, seed weight), and ultimately, the grain nutrient profile (N, K, Na) under defined irrigation regimes (40% vs. 80% field capacity).

## Results

**Fig. 1:**
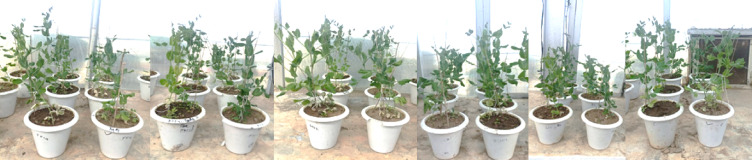
Pot trail on Pea plant under green house condition.

The influence of PGP microbial inoculants on pea growth and yield attributes under normal irrigation (80% FC) and drought stress (40% FC) was evaluated through a pot experiment (Fig. 1). Significant variations were observed among treatments for all measured parameters.

### Impact of PGP inoculants on RWC and proline accumulation and Na/K ratio in pea under drought stress

The results shows the effect of PGP microbial inoculants (FM20, FM65, FM19, W22, OF5, and Control) combined with drought stress (40% FC) and normal irrigation (80% FC) on three physiological stress indicators: RWC, proline accumulation (µmole/gFW) and Na/K ratio in straw and grain.

**Fig. 2:**
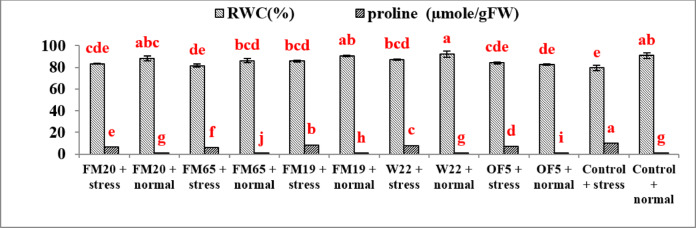
Bar diagram to show RWC and proline content in *Pisum sativum.*

**Fig. 3:**
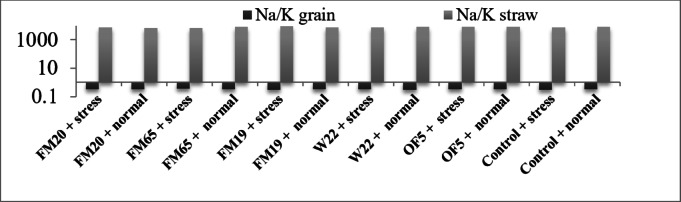
Bar diagram to show Na/K raio in straw and grain in *Pisum sativum.*

- RWC: It indicates the water status of the pea leaves. Higher RWC is desirable, especially under stress. The highest RWC (94.3%) was observed in the W22 + normal treatment. All normal irrigation treatments maintained a high RWC . The lowest RWC was observed in the Control + stress treatment (81.7%), confirming that unmitigated drought severely compromised the plant’s water balance. Inoculants significantly mitigated the drop in RWC caused by drought. For instance, the W22 + stress treatment maintained RWC at approximately 88.5%, which was similar to most of the normal irrigation treatments but significantly higher than the Control + stress treatment. Similarly treatments FM19 + stress (87.0%) and FM65 + stress (85.0%) also showed a statistically significant improvement over the uninoculated control under stress (Fig. 2).

b. Proline content: Proline (µmole/gFW) is an osmotic regulator that accumulates in plants under stress. Higher proline accumulation indicates greater stress tolerance. The highest proline accumulation was recorded in the FM65 + normal treatment (10.5 µmole/gFW), followed by FM19 + stress (10.0 µmole/gFW) and lowest accumulation was found in the W22 + normal treatment (5.7 µmole/gFW). Drought stress consistently induced higher proline accumulation across all treatments compared to their non-inoculated, normal-irrigated counterparts. The data suggests that the presence of the PGP inoculants, especially FM65 and FM19, triggered a robust osmotic adjustment mechanism, as shown by their high proline levels, regardless of irrigation level (FM65 + normal irrigation). High proline accumulation under stress indicates the inoculant helps the plant cope by activating this key defence mechanism (Fig. 2).

c. Na/K ratio in straw: The primary objective was to assess which bacterial isolates could significantly enhance ionic homeostasis under the drought condition (40% FC). Efficacy was determined by comparing the straw Na/K ratios of inoculated plants against the stressed uninoculated Control (8656.716). Microbial inoculation resulted in highly strain-specific effects, identifying superior ameliorators across the tested panel (Fig. 3; Table 1).

**Table 1:** Percent change of Na/K ratio in straw part of different treatments w.r.t control.

Bacterial Isolate	Straw Na/K Ratio	Absolute Difference from Control	% Change (Amelioration/Detriment)
FM20 + 40% FC	7165.109	− 1491.607	− 17.23% (Superior)
FM20 + 80% FC	6567.164	− 1887.646	− 22.33% (Strong Constitutive Effect)
FM65 + 40% FC	7317.073	− 1339.643	− 15.47% (Superior)
FM65 + 80% FC	7476.636	− 978.174	− 11.57% (Moderate Constitutive Effect)
FM19 + 40% FC	8615.385	− 41.331	− 0.48% (Minimal)
FM19 + 80% FC	7356.948	− 1097.862	− 13.09% (Moderate Constitutive Effect)
W22 + 40% FC	7536.232	− 1120.484	− 12.94% (Strong)
W22 + 80% FC	7917.889	− 536.921	− 6.44% (Minor Constitutive Effect)
OF5 + 40% FC	8405.797	− 250.919	− 2.90% (Marginal)
OF5 + 80% FC	7848.837	− 605.973	− 7.16% (Minor Constitutive Effect)
Control + 40% FC	8656.716	0 (Baseline)	0
Control + 80% FC	8454.81	0 (Baseline)	0

Isolate FM20 demonstrated the most superior performance in managing ionic stress under drought. It reduced the straw Na/K ratio to 7165.109, representing a 17.23% reduction relative to the stressed control (8656.716). This substantial decrease suggests that FM20 efficiently enhances the pea plant’s endogenous stress response mechanisms, likely by boosting K absorption (potentially via AtHKT1-like signaling) or by increasing the efficiency of Na exclusion at the root-shoot interface (Table 1).

Isolate FM65 was the second most effective, achieving a highly significant 15.47% reduction, lowering the straw Na/K ratio to 7317.073. W22 also provided a strong ameliorative effect, with a 12.94% reduction (7536.232). OF5 showed marginal benefit (−2.90%), and FM19 offered only a minimal reduction (−0.48%). These data confirm that successful PGPR strains can fundamentally stabilize the systemic ionic balance in *P. sativum* under water deficit (Table 1).

The reproductive tissue demonstrated exceptional ionic stability, with Na/K ratios tightly clustered between 0.310345 and 0.342857. In the uninoculated control, drought stress (40% FC, 0.324786) caused a slight but measurable increase in the Na/K ratio compared to the well-watered condition (80% FC, 0.310345), indicating that drought slightly compromises the plant’s ability to exclude Na from the developing grain.

### Impact of PGP inoculants on plant health through shoot and root length

Under drought stress, the longest shoots were recorded in FM65 + stress (61.67 cm) and FM20 + stress (55 cm), both statistically similar to several other inoculated treatments under normal irrigation condition. Under normal conditions, FM20 + normal and FM65 + normal exhibited the maximum shoot length (63.33 cm). The lowest shoot length was observed in the Control + stress (43.33 cm). Overall, inoculated treatments maintained shoot length under stress compared to the uninoculated control. The shoot length of Control + stress (43.333 cm) was statistically lower than all other treatments, confirming the severity of the drought stress and the effectiveness of all inoculants in mitigating this effect to some degree (Table 2).

**Table 2:** Plant health parameters.

	Treatments	*Average shoot length (cm)	*Average root length (cm)	*Average pod length (cm)	*Average No. of pods	*Average pod weight (g)
1	FM20 + stress	55^abc^ ± 2.887	17^ab^ ± 1.528	6.148^bc^ ± 0.343	11.333^b^ ± 0.667	7.243^c^ ± 0.46
2	FM20 + normal	63.333^a^ ± 1.667	11.333^cd^ ± 2.728	6.553^abc^ ± 0.094	14^b^ ± 2.309	8.177^c^ ± 0.394
3	FM65 + stress	61.667^ab^ ± 6.009	12^bcd^ ± 1.155	5.519^bc^ ± 0.305	16^b^ ± 2	14.067^b^ ± 1.084
4	FM65 + normal	63.333^a^ ± 4.41	12.333^bcd^ ± 2.333	6.954^abc^ ± 0.511	22.333^a^ ± 2.603	19.347^a^ ± 0.663
5	FM19 + stress	52.667^abc^ ± 2.667	12^bc±d^ ± 1.528	5.476^bc^ ± 0.363	11.667^b^ ± 3.18	8.7^c^ ± 1.438
6	FM19 + normal	55.667^abc^ ± 6.36	12.167^bcd^ ± 1.167	7.187^ab^ ± 0.032	11.333^b^ ± 2.028	8.593^c^ ± 0.257
7	W22 + stress	55^abc^ ± 2.887	13^abcd^ ± 1.528	7.111^ab^ ± 1.06	13.667^b^ ± 1.667	9.06^c^ ± 0.645
8	W22 + normal	51^bc^ ± 2.082	17^ab^ ± 2.517	8.058^a^ ± 0.603	13.333^b^ ± 1.202	11.113^b^ ± 1.655
9	OF5 + stress	52.333^abc^ ± 4.333	18.333^a^ ± 0.882	6.352^bc^ ± 0.103	11.667^b^ ± 1.202	11.63^a^ ± 1.79
10	OF5 + normal	56.667^ab^ ± 1.667	16.667^abc^ ± 1.202	6.96^abc^ ± 0.506	13.667^b^ ± 0.882	10.697^b^ ± 1.752
11	Control + stress	43.333^c^ ± 1.667	14^abcd^ ± 1	5.245^c^ ± 0.487	10^b^ ± 0.577	8.073^c^ ± 0.633
12	Control + normal	50^bc^ ± 2.887	10^d^ ± 1.155	6.369^bc^ ± 0.694	11.667^b^ ± 0.333	4.63^d^ ± 1.594
	C.D	10.707	4.904	1.489	5.202	3.441
	SE(m)	3.646	1.67	0.507	1.772	1.172
	SE(d)	5.157	2.362	0.717	2.506	1.658
	C.V	11.483	20.933	13.524	22.919	20.078
	C.D	10.707	4.904	1.489	5.202	3.441

*Significance < 0.001.

Root length varied significantly across treatments, ranging from 10 cm (Control + normal) to 18.33 cm (OF5 + stress). Under drought stress, OF5 showed the highest root length, followed by FM20 and W22. Under normal conditions, W22 + normal (17 cm) and OF5 + normal (16.67 cm) showed superior root length. Most inoculated treatments outperformed the control in both moisture regimes (Table 2). OF5 + stress were significantly superior to many other treatments, including Control + normal and FM20 + normal. Interestingly, the highest root lengths were generally found in the stress-treated groups (OF5 + stress and FM20 + stress), potentially as an adaptive response facilitated by the PGP inoculant (Table 2).

### Impact of PGP inoculants on Pod yield

Pod length was significantly highest in W22 + normal (8.06 cm) and FM19 + normal (7.19 cm). Under stress, pod length declined in all treatments, with the lowest value recorded in Control + stress (5.25 cm). However, inoculated treatments such as W22 + stress (7.11 cm) and OF5 + stress (6.35 cm) maintained comparatively higher pod length under drought. The highest average number of pods was recorded under FM65 + normal (22.33 pods), which was significantly superior to all other treatments. Under drought stress, the number of pods ranged from 10 (Control + stress) to 16 (FM65 + stress). Inoculated treatments generally produced more pods than the control under both moisture levels, indicating improved pod formation due to microbial inoculation. Pod weight varied markedly among treatments. Statistical grouping indicates that only the FM65 + normal treatment was significantly different from the rest. All other 11 treatments fell into the same statistical group (‘b’) for this parameter, suggesting a wide range of inoculants offered a similar, moderate improvement over the absolute control under stress. The maximum pod weight was observed in FM65 + normal (19.35 g), followed by OF5 + stress (11.63 g) and W22 + normal (11.11 g). Under drought, most inoculated treatments produced higher pod weight compared to Control + stress (8.07 g). Under normal irrigation, pod weight was lowest in Control + normal (4.63 g). Across all measured parameters—shoot and root growth, pod formation, and pod weight PGP microbial inoculants consistently outperformed the uninoculated control under both drought stress and normal irrigation. In particular, FM65, OF5, and W22 demonstrated strong potential to alleviate drought-induced yield reduction in pea (Table 2). Despite the large numerical differences, the statistical analysis grouped all treatments into the same letter group (‘a’) for pod weight. This suggests that while there were large quantitative differences, the variability within replicates for this parameter was high, or the Critical Difference value (3.441) was large enough that no two treatments were statistically distinct at the chosen level of significance. This result should be interpreted cautiously, but numerically, FM65 clearly provided the highest yield-related metrics under both normal and stress conditions.

### Impact of PGP inoculants on pea seed weight parameters under drought stress

The provided results illustrates the effect of various PGP microbial inoculants (FM20, FM65, FM19, W22, OF5, and Control) combined with two irrigation regimes (stress at 40% FC and normal at 80% FC) on two key yield parameters: seed weight per plant (g) and 100 seed weight (g).

**Fig. 4:**
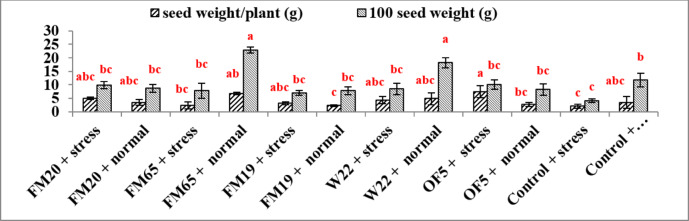
Bar plot to represent the impact of different treatments on seed yield different superscript letter (a,b,c)represent significant difference between groups at p less than 0.05.

Seed weight per plant is a direct measure of productivity. The maximum seed weight per plant (6.7 g) was achieved under FM65 + normal irrigation. The results indicate under stress conditions, the PGP inoculants helped to maintain seed yield significantly above the control. The highest seed weight under drought stress was recorded in the OF5 + stress treatment (7.3 g), which was statistically similar to the yields of several normal-irrigated treatments. On the other hand lowest seed weight was observed in the Control + stress treatment (2.0 g), highlighting the impact of unmitigated drought (Fig. 4).

The 100 seed weight indicates the average seed size and quality. The largest seed size was found in the FM65 + normal treatment, reaching the maximum value (22.9 g), which was statistically distinct from most other treatments. Drought stress significantly reduced seed size in the non-inoculated control. The Control + stress treatment showed the lowest 100 seed weight (3.2 g). Inoculation with W22 + stress resulted in the largest seed size under drought stress (8.7 g), although statistically similar to several other stress and normal treatments. The FM65 + normal treatment achieve the highest 100 seed weight followed by FM20 + normal (9.9 g). The FM65 inoculant was the most effective treatment for maximizing both seed weight per plant and 100 seed weight under normal irrigation. Under drought stress, the OF5 inoculant provided the highest numerical seed weight per plant, and the W22 inoculant provided the highest numerical 100 seed weight, demonstrating a strain-specific ability to mitigate drought effects on different yield parameters. All inoculated treatments generally resulted in statistically superior or similar performance compared to the non-inoculated Control + stress treatment, highlighting the general benefit of PGP application in ameliorating drought-induced yield losses (Fig. 4).

### Impact of PGP bioinoculants on nutrient status of straw and grain of pea

#### Nitrogen partitioning

**Fig. 5:**
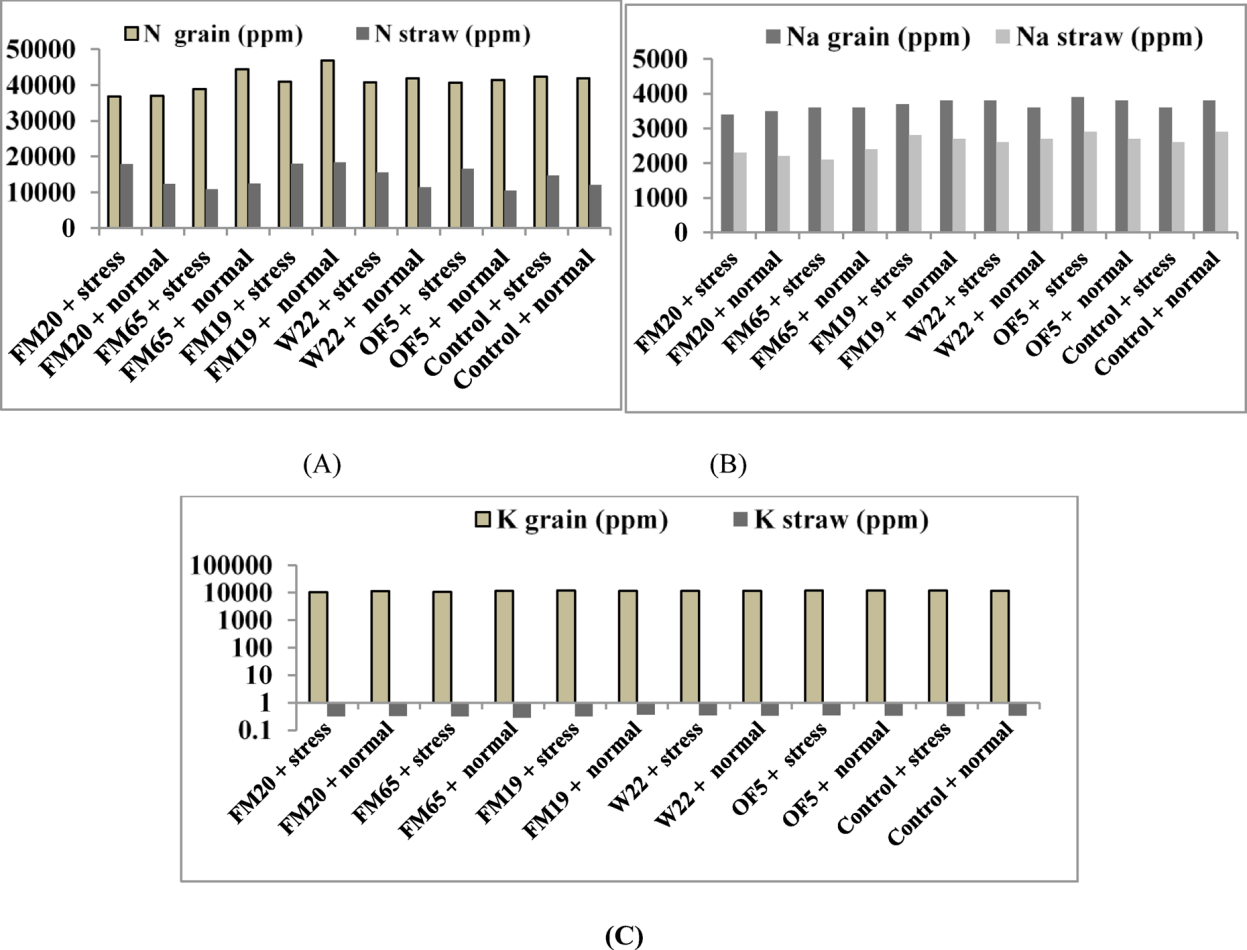
Bar diagram representing (**A**) N content (**B**) Na content (**C**) K content in grain and straw.

Nitrogen (N) is a critical macronutrient, fundamental to plant growth, photosynthetic activity, and reproductive development. Successful N partitioning the efficient mobilization of N compounds from vegetative tissues (straw) into the reproductive sink (grain) is paramount for determining grain yield and quality. In uninoculated control the nitrogen in grain showed a minimal increase under drought (42,334 ppm) compared to optimal watering (41,893 ppm), suggesting that the plant prioritized the concentration of existing N resources into the reproductive sink potentially due to biomass dilution effect. But on the other hand N in straw increased sharply by 22.33% under drought (14,741 ppm) compared to normal conditions (12,051 ppm). This accumulation suggests that drought stress impaired N remobilization efficiency, possibly due to reduced proteolytic activity associated with water deficit. Under drought stress, all isolates resulted in a reduction of N in grain compared to the control, suggesting that the inoculation may have negatively impacted N assimilation or remobilization kinetics under extreme water deficit. FM20 showed the largest detrimental effect, reducing N in grain by -12.99%. However, in terms of N remobilization efficiency, isolate FM65 was the sole strain to significantly improve performance, reducing residual N in straw -26.04%. This indicates that FM65 successfully enhanced the plant’s efficiency in transferring N from vegetative storage to the grain sink, overcoming the stress-induced impairment observed in the control. Under normal irrigation conditions, FM19 demonstrated the superior N acquisition and partitioning capability, resulting in a + 11.71% increase in N-grain content. FM65 also provided a significant benefit (+ 6.01%). The high N-grain content is indicative of successful PGPR mechanisms, such as enhanced nitrogen fixation or improved nutrient solubilization, which increase the overall N pool available to the plant (Fig. 5a).

#### Na content and exclusion efficiency

In uninoculated control, drought stress (40% FC) caused an increase in both Na-grain (3800 ppm vs 3600 ppm) and Na-straw (2900 ppm vs 2600 ppm) compared to optimal irrigation (80% FC). This confirms that water deficit leads to an undesirable concentration and increased accumulation of Na in both vegetative and reproductive tissues. Isolate FM65 demonstrated the most potent vegetative Na exclusion mechanism, reducing Na-straw content by − 27.59%. FM20 also exhibited strong performance, reducing Na-straw by − 20.69%. FM20 achieved the highest reduction in Na-grain content (− 10.53%), confirming it as the best absolute Na excluder from the reproductive sink under stress. FM65 and FM19 offered less effective grain Na exclusion relative to FM20. OF5 failed to exclude Na, resulting in a slight increase (+ 2.63%) in Na-grain (Fig. 5b).

#### K content and aquision efficiency

Potassium (K) is vital for maintaining turgor, enzyme activation, and balancing osmotic potential, making its successful acquisition and partitioning a key indicator of PGPR mediated drought tolerance. In the uninoculated Control plants, drought stress (40% FC) resulted in a minimal increase in K-grain (11,700 ppm vs 11,600 ppm) compared to optimal watering (80% FC), suggesting that the plant attempts to maintain grain K-grain concentration even under water deficit. Conversely, K-straw remained stable at 0.335 in both conditions.

Under stressed condition, isolate FF19 (1.71%) was only treatment responsible for increase acquisition of K ions in grain whereas OF5 (2.99%) and W22 (2.99%) increased K uptake in grain. On the other hand in case of normal irrigation condition OF5 (1.72%) showed increase K acquisition in grain and FM19 (9.55%) in case of straw in comparison to control (Fig. 5c).

#### Principal component analysis (PCA) and correlation matrix

**Fig. 6:**
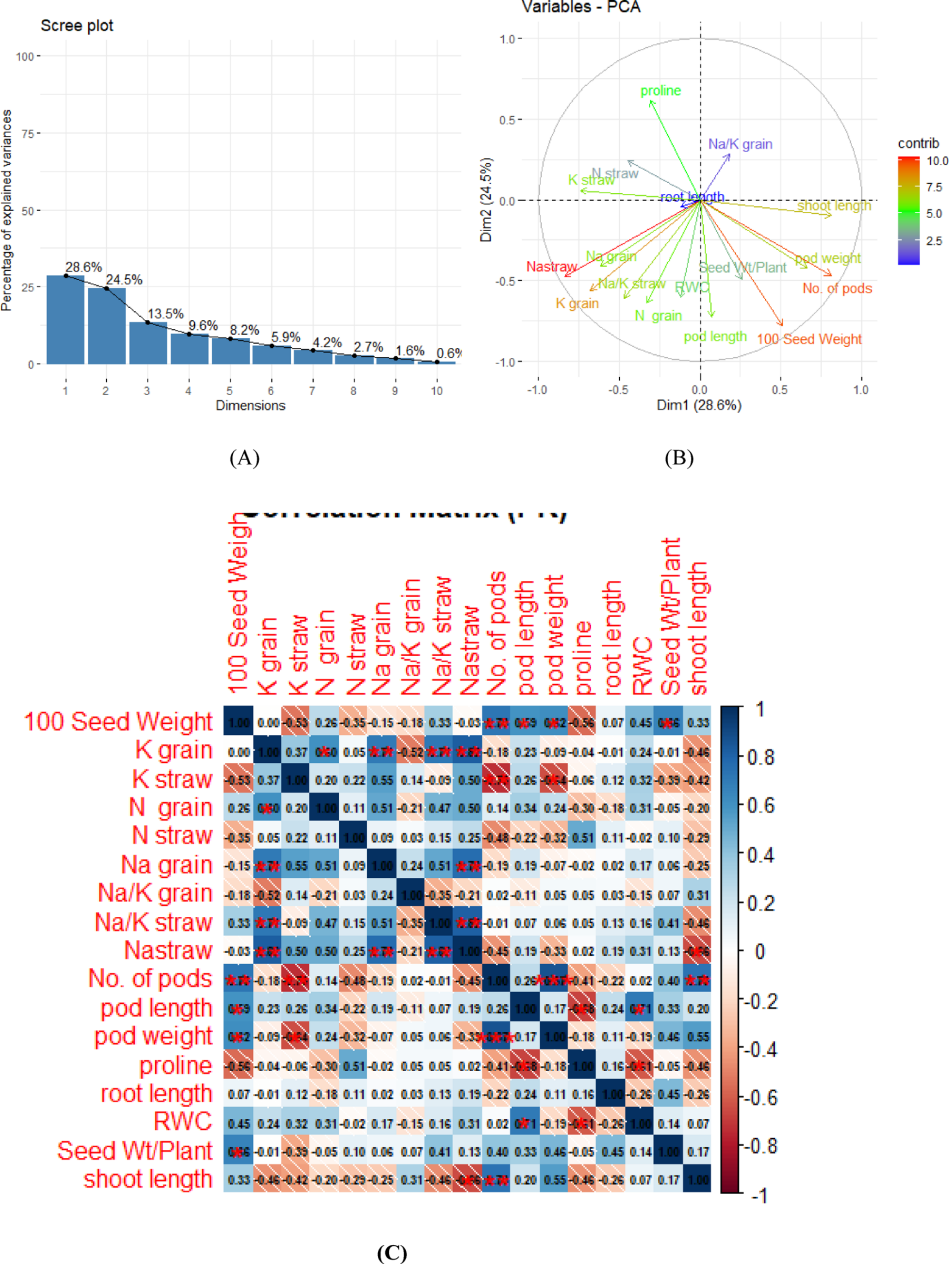
(**A**): Scree plot to show Eigen values (**B**) Biplot analysis (**C**) Correlation matrix, where single star shows significance at 0.01%. ^*^level of significance was set at 0.001, 0.01 and d 0.05.

Variables like shoot length, pod weight, No. of pods, 100-Seed Weight, pod length, and Seed Wt/Plant are clustered together and point towards the positive side of Dim1. This suggests they are all positively correlated with each other (e.g., higher pod weight is associated with a higher number of pods and higher 100-seed weight). This group represents the enhanced vegetative growth is tightly linked to reproductive output. This cluster represents the yield potential and yield stability axis, reflecting coordinated biomass production and effective source–sink relationships. Variables like Na-straw, K grain; Na grain, Na/K straw, and N grain are grouped in the bottom-left quadrant. They are generally correlated with each other. But Bottom Right is negatively correlated with the Stress/Ionic Group 1 (Bottom Left) and Na-straw in particular. This is common in stress studies: traits related to plant yield decrease as certain stress-related variables (like high Na accumulation in straw) increase. The inverse relationship between plant productivity and Na accumulation reflects a yield–ionic stress trade-off where increased Na accumulation in vegetative tissues is associated with reduced reproductive performance. Proline (Top Left) appears to be relatively uncorrelated with the main Yield/Growth Group (Bottom Right), as they form an angle greater than 90 °C. While proline accumulation reflects stress perception and cellular protection mechanisms, it does not necessarily translate into improved growth performance (Fig. 6A, B).

The correlation analysis shows all the yield parameters are highly correlated with each other and Na is a yield inhibitor as it negatively correlated with all yield contributors and proline acted as good stress indicator positively correlated with stress indicators and some yield factors as well (Fig. 6C).

## Discussion

The results demonstrate a dual role for PGP inoculants in mitigating drought stress at the physiological level. PGP inoculation dramatically improved the plant’s water status and activated key osmotic defence mechanisms. Inoculation with W22 + stress resulted in an RWC of 88.5%, representing an improvement of approximately 8.3% compared to the Control + stress treatment (81.7%). Similarly Bio-inoculants have been shown to improve drought tolerance in barley, leading to higher leaf relative water content compared to uninoculated plants^17^. As per the literature available the ability of PGP to improve water use efficiency (WUE) and maintain cell turgor under stress is crucial for sustaining metabolic functions, often through the production of exopolysaccharides that bind soil water^18–20^. FM19 under stress treatment induced a high proline accumulation (25% higher) in comparison to than the Control in stressed condition. This elevated proline acts as an efficient osmoprotectant and molecular chaperone, safeguarding cellular integrity and metabolic enzymes from dehydration-induced damage, a known mechanism amplified by microbial signalling. Similar results were reported in rice^21^, Faba Bean (*Vicia faba*) along with Pea (*Pisum sativum*)^22^, sorghum^23^ and wheat^24^ who observed accumulation of osmoprotectants such as proline during water stress.

In case of Na/K ratio levels FM20 was identified as the most effective overall inoculant for vegetative health, performing optimally under both well-watered (-22.33% reduction) and drought conditions (-17.23% reduction) in the straw. FM65 was identified as a strong ameliorator for vegetative tissue, providing significant benefit under stress (−15.47% reduction) and normal condition (-11.57% reduction). In contrast, FM19 demonstrated a novel specialization: while providing minimal vegetative amelioration (-0.48% reduction in straw under stress), it was the most successful isolate at maintaining the lowest Na/K ratio in the grain (0.310924) under drought stress. This comparative analysis of Na/K ratio in straw and grain highlights a crucial physiological trade-off. PGPR isolates that are highly effective at promoting Na sequestration in the straw (FM65 and FM20) appear to marginally increase the absolute level of Na passing the vegetative barriers and reaching the grain. These results suggest that different PGPR strains specialize in different compartments of the ionic defence mechanism. FM65 and FM20 are highly effective vegetative-ionic stress ameliorators, while FM19 is an effective reproductive-ionic exclusion specialist under stress. According to Cakmak et al.^25^ adequate K status improve stress tolerance through enhanced water use efficiency and better growth and development in plant. Similarly Subbarao et al.^26^ and Krishnasamy et al.^27^ also reported improved Eucalyptus growth in water stress through Na replacement by K.

An improvement in shoot length (42.3%) in case of FM65 treated plants in comparison to control under stress condition was observed. This strong vegetative growth recovery is indicative of the PGP inoculant’s capacity to synthesize phytohormones like auxins and gibberellins, which overcome the growth inhibition typically imposed by high ABA levels under drought^28^. Shoot elongation is associated with higher auxin production by microbial inoculants which improve the nutrient uptake even under stressed condition^28,29^. A significant increase in pod length of approximately 35.4% was recorded due to W22, 60% increase in pod number in FM65 treatments and 44.1% increase in pod weight by OF5 over the control in stressed conditions. The numerical superiority of FM65 under normal conditions and the strong performance of W22 and OF5 under stress highlight their potential as effective bio-inoculants for pea cultivation. The study results align with global research trends confirming that PGPR provide a sustainable, cost-effective tool to minimize crop losses under limited water availability and to boost baseline productivity, representing a significant stride toward drought-resilient agriculture^29^.

Maximum seed weight per plant in stressed condition was recorded in OF5 with 265% increase with respect to control under similar condition. This demonstrates the inoculant’s capacity to sustain high carbon fixation and translocation to the seeds even under limiting water conditions. But 100 seed weight which is an indicator of seed quality was maximum in W22 with 172% increase in comparison to control under stressed conditions. Similar enhancement in yield of maize plants was observed after the treatment with microbial bioinoculants^30–32^. A significant yield protection in FM 65 treated stressed condition (60% increase with respect to control under similar condition) suggests that the treatment maintained resource flow and reduced the stress-induced flower/pod abortion rate, a critical factor during the reproductive stage. The preservation of seed quality is likely due to the PGP-induced delay in senescence, prolonging the effective period for photosynthesis and nutrient accumulation in the seeds.

The variable responses of the inoculants underscore the intricate nature of microbe–plant interactions under drought conditions and reaffirm that PGPR-mediated drought tolerance is highly strain-dependent. These findings align with earlier reports demonstrating that PGPR can alleviate drought-induced damage by enhancing nutrient-use efficiency, sustaining ionic balance, and improving nutrient remobilization, although their effectiveness often varies with the severity of stress and compatibility with the plant host. A positive correlation between soil moisture status and applied potassium levels has been reported by Nandwal et al.^33^. Likewise, Achakzai^34^ and Lazof and Cheeseman^35^ showed that variations in water potential significantly influenced the uptake and accumulation of cations such as Na and K in different crops. The most compelling finding is the clear trade-off between yield potential and Na accumulation, which constitutes the major source of variation. This strongly suggests that Na exclusion and/or compartmentalization are the critical physiological determinants of stress tolerance, as plants that limit Na transport to the straw (Na-straw) are able to maintain higher Seed Wt/Plant and 100-Seed Weight. This aligns with studies indicating that maintaining a favourable K/Na ratio is essential for enzyme function and protein synthesis in the cytosol, underpinning growth and yield stability in saline environments^36,37^.

The role of proline as an independent component in the PCA and its mild positive correlation with yield factors suggests it is an active osmoprotectant rather than merely an indicator of tissue damage. Proline likely functions to stabilize membranes and macromolecules, contributing to the maintenance of RWC and overall plant performance under stress, highlighting a key aspect of metabolic resilience^38,39^.

Overall, the findings demonstrate that PGPR inoculation enhances drought resilience in pea by improving water status, maintaining ionic balance and maintaining yield related traits. Since the study was conducted under controlled pot conditions the responses may differ under actual field condition depending upon soil heterogeneity, microbial competition and fluctuating climatic factors. Therefore field-based validation is necessary before recommending large-scale application.

## Material and methods

### Bacterial inoculants and plant treatment

**Fig. 7:**
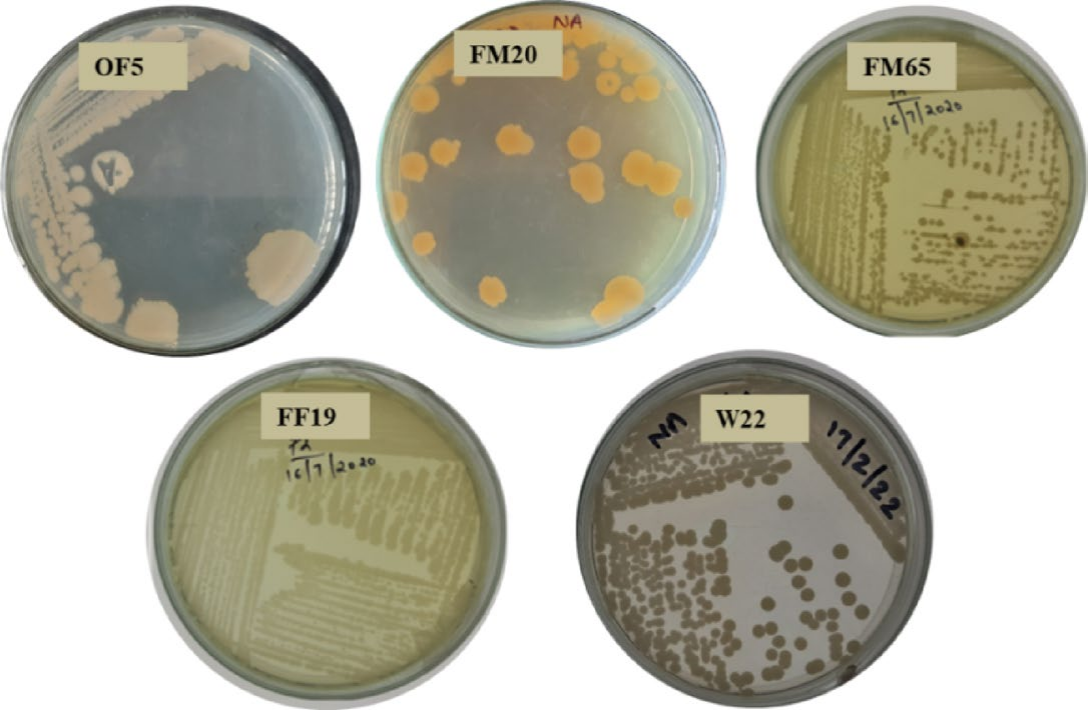
Plate morphology of bacterial isolates.

The bacterial inoculants were good plant growth promoters (Zn, P solubilization, siderophore production and drought stress tolerance in PEG-6000). Two strains of *Priestia, Priestia megaterium*-W22 (Accession No: *OR880303)* and *Priestia megaterium*-FM 65 (Accession No: *OR880333)* are evaluated along with three other isolates recovered from wheat (OF5) and finger millet (FM20 and FM19)^16^ (Fig. 7). Inoculum of each bacterial strain was prepared by growing them separately on nutrient agar for 48 h at 28 °C. Cells were collected from the agar plates and washed three times into 1 mL using sterilized deionized water. Optical density of the cells was adjusted to OD600 of 1.0 in 10 mL of sterile water which is equivalent to 1 × 10^7^ CFU ml^−1^. Seeds of peas were surface sterilized using 10% (v/v) of sodium hypochlorite for 2 min twice. The seeds were then immersed in the bacterial cell suspension for 3 h without agitation. As a control, some seeds were immersed only in sterile water without bacterial cells.

### Field capacity management

Pots with 5-L capacity randomly selected and filled with 5 L of soil mixture. The soil was thoroughly watered upto saturation and weighed. The soils were then allowed to sit for 3 days after excess soil saturation in darkness and covered in poly bags in order to prevent evaporation. The weight of after 3 days was used to calculate soil water holding. The soils were then dried for 7 days in an oven at 70 °C, until complete dryness. Data derived from the above soil water moisture status were then used to compute estimates of the gravimetric water content and the amount of water needed to be added to establish specific field capacities^40^.

### Pot experimentation

The experiment was designed with completely randomised design in three replication under controlled temperature (32 ± 2 °C) and relative humidity (40 to 50%) in November 2024 at ICAR-VPKAS, Almora, Uttarakhand, India. The treatments are mentioned in Table 3. The soil was put into 5-cm-wide, 25-cm-tall pots. 5 inoculated seeds were sown into each pot and which were thinned to three after 15 days. Irrigation to the pots was done based on field capacity (40% for stressed treatments and 80% for normal treatments).

**Table 3:** Different treatments in field trial.

S. No	Treatment	Details
1	FM20 + stress	FM20 in 40% field capacity of irrigation
2	FM20 + normal	FM20 in 80% field capacity of irrigation
3	FM65 + stress	FM65 in 40% field capacity of irrigation
4	FM65 + normal	FM65 in 80% field capacity of irrigation
5	FM19 + stress	FF19 in 40% field capacity of irrigation
6	FM19 + normal	FF19 in 80% field capacity of irrigation
7	W22 + stress	W22 in 40% field capacity of irrigation
8	W22 + normal	W22 in 80% field capacity of irrigation
9	OF5 + stress	OF5 in 40% field capacity of irrigation
10	OF5 + normal	OF5 in 80% field capacity of irrigation
11	Control + stress	Un-inoculated in 40% field capacity of irrigation
12	Control + normal	Un-inoculated in 80% field capacity of irrigation

### Plant physio-chemical properties

#### Relative water content

Relative water contents was measure on fresh weight (0.5 g) basis. Leaves were soaked in double distilled water for 24 h. Turgid weight was measured after carefully removing the water on the leave’s surface. To measure leave dry weight, leave samples were dried in an oven for 24 h at 80 °C. The RWC were calculated as per the formula:

RWC = (FW-DW) / (TW-DW) × 100 Where FW is the fresh weight of sample, DW is the dry weight of sample and TW is the turgid/soaked weight of sample by Karrou and Maranville,^41^.

#### Proline content

The proline content was estimated following the method by Bates et al.^42^. The plant tissue (0.5 g) was homogenised in 3% aqueous sulfosalicylic acid which was further centrifuges at 9000 rpm for 10 min at 5° C. The supernatant (2 ml) was mixed with 2 ml of acid ninhydrin and 2 ml of glacial acetic acid mixture and kept in boiling water bath for 1 h. The mixture was shifted to ice bath to stop the reaction and 4 ml of toluene was used to extract the toluene which was recorded through absorbance at 520 nm. The proline content was measured using the formula:

$${\mathrm{Proline}}\,{\text{content }}\left( {{\mu g}/{\mathrm{ml}}} \right) = \frac{{\left[ {A_{520} \times D} \right]}}{0.0057}$$

### Plant health parameters

#### Plant root and shoot length

Plant and shoot length (cm) was measured from the soil surface to the tip using a meter scale at harvesting stage. The mean was recorded and used for analysis.

#### Average pod length, average no. of pods per plant and average pod weight

Total number of pods per plants was counted and average of all three plants per pots was estimated to calculate Average No. of pods per plant. Average pod length and average pod weight was calculated by estimating length and weight per pods and taking an average of all pods per pot.

### Yield components

#### Average seed weight per plant

Total seed weight per plant was estimated and an average of all there plants per pot was calculated.

#### 100 seed weight

Total seed weight of each pot was used to calculate 100 seeds weight using the formula.

$${\mathrm{Hundred}}\,{\mathrm{seed}}\,{\mathrm{weight}} = \left( {{\mathrm{Total}}\,{\mathrm{seed}}\,{\mathrm{weight}}/{\mathrm{no}}\,{\mathrm{of}}\,{\mathrm{total}}\,{\mathrm{seeds}}} \right)*{1}00$$

### Nutrient analysis of grains

The grains from three replicated were pooled into one sample. The dried grains were cleaned manually to remove any broken or foreign materials. The samples were combined into one composite (pooled) sample before analysis, the laboratory measurement represents the average nutrient composition of that combined material rather than independent biological replicates. Cleaned samples were then oven-dried at 70 °C for 48 h to eliminate residual moisture. Dried grains were ground using pestel and mortar. For Na and K mixture of three acids (Nitric Acid, Perchloric Acid and Sulfuric Acid) was used to digest the samples in microwave digestion system (MARS) and digested clear solution was analysed for K and Na in Flame Photometer (Model 1385)^43,44^.

Similarly for total nitrogen grounded samples were weighed and digested Concentrated H2SO4 is added. This acts as an oxidizing agent to break down the organic matter and a reducing agent for the nitrogen, converting it into ammonium sulphate.

A catalyst tablet is added to speed up the reaction and increase the boiling point of the acid. It typically contains Potassium Sulfate and Copper Sulfate. The tubes are placed in a Kjeltec block digestor and heated systematically. Heating continues for several hours until the black, charred solution becomes clear and translucent. This indicates all organic nitrogen has been converted to ammonium sulphate. Alkalization of ammonium sulphate leads to formation of ammonia which is titrated along with boric acid in HCL to determine the amount of total N%. Crude protein % is estimated by multiplying the %N with a factor (5.7 for Pea seed and 6.25 for Pea straw)^45^.

### Statistical analysis

The data in 3 replication was statistically analysed through Anova using OPSTAT. Post hoc analysis was done using Duncan’s Multiple range test. The *P* value was < 0.001. The PCA analysis was done to study the trend and correlation of different treatments with different parameters selected. Principal Component Analysis (PCA) was performed using the measured agronomic and ionic traits, including shoot length, pod weight, number of pods, 100-seed weight, pod length, seed weight per plant, Na-straw, Na-grain, K-grain, Na/K-straw ratio, N-grain, and proline content. Variables were selected based on their biological relevance to yield performance and salt stress response. All the data was also mean centred for normalization.

## Data Availability

The bacterial isolates are well characterized and submitted in NCBI with accession code *OR880303* (W22); *OR880333* (FM65). The data is also provided within the related files which contain a previous publication by corresponding author.

## Abbreviations

FC: Field capacity
PGPR: Plant growth-promoting rhizobacteria
RWC: Relative water content
PCA: Principle component analysis
WUE: Water use efficiency
PEG: Polyethylene glycol
FW: Fresh weight
DW: Dry weight
TW: Turgor weight
OPSTAT: Operational statistics

## Declarations

### Dual publication

The results/data/figures in this manuscript have not been published elsewhere, nor are they under consideration (from you or one of your Contributing Authors) by another publisher.

### Third party material

All of the material is owned by the authors and/or no permissions are required.
